# Role of Ca^2+^ in changing active force during intermittent submaximal stimulation in intact, single mouse muscle fibers

**DOI:** 10.1007/s00424-018-2143-y

**Published:** 2018-04-18

**Authors:** Lisa D. Glass, Arthur J. Cheng, Brian R. MacIntosh

**Affiliations:** 1 Human Performance Laboratory, Faculty of Kinesiology, University of Calgary, Calgary, Alberta T2N 1N4 Canada; 2 Department of Physiology and Pharmacology, Karolinska Institutet, SE-171 77 Stockholm, Sweden

**Keywords:** Fatigue, Potentiation, Calcium sensitivity, Half-maximal calcium concentration, Staircase

## Abstract

Fatigue of single mouse fibers during repeated high-frequency stimulation results initially from decreased Ca^2+^ sensitivity while free myoplasmic calcium concentration ([Ca^2+^]_m_) increases, followed by decreasing [Ca^2+^]_m_. Recovery of active force with low-frequency stimulation is slow and persistent fatigue results from low [Ca^2+^]_m_. However, the consequences of intermittent submaximal contractions are not known. The aim of the present study was to investigate the changes in [Ca^2+^]_m_ and active force during intermittent submaximal contractions and subsequent recovery. Single fibers of mouse flexor digitorum brevis muscles at 32 °C were stimulated with 40 or 50 Hz, for 350 ms every 2 s for 2 min and then every 1 s until < 40% of initial force. Values obtained during the intermittent stimulation were compared with a control force-[Ca^2+^]_m_ relationship. A “P”-shaped pattern in the force-[Ca^2+^]_m_ relationship was observed during intermittent stimulation. Early in the intermittent stimulation, [Ca^2+^]_m_ increased while active force decreased. Subsequent force potentiation was accompanied by increased Ca^2+^ sensitivity. Later, as active force declined, [Ca^2+^]_m_ decreased significantly (*p* < 0.001). This was followed, in the final phase, by a significant decrease in Ca^2+^ sensitivity determined by [Ca^2+^]_m_ at half-maximal force (Ca_50_) (*p* = 0.001). Low-frequency fatigue persisted during recovery while Ca_50_ was not significantly different from prefatigue (*p* > 0.5). In conclusion, the main mechanism of fatigue is due to decreases in both [Ca^2+^]_m_ and Ca^2+^ sensitivity following the initial force potentiation. The intermittent submaximal contractions resulted in persistent low-frequency fatigue seen during recovery, which was explained by depressed [Ca^2+^]_m_ with no change in Ca^2+^ sensitivity.

## Introduction

The force exerted by a muscle expressed relative to myoplasmic free Ca^2+^ concentration ([Ca^2+^]_m_) gives a sigmoidal relationship [1, 2]. Observations of contractions under different conditions (frequency of stimulation, activity-dependent potentiation, force depression due to prior activation, weakness due to aging or disuse) can effectively be considered on the force-[Ca^2+^]_m_ relationship to provide clues to the underlying mechanisms effecting change in active force [3]. A change in active force results from one of the following: a change in [Ca^2+^]_m_, a change in Ca^2+^ sensitivity, or a change in maximal force generating capacity [4, 5]. Ca^2+^ sensitivity can be represented by a change in half-maximal [Ca^2+^] (Ca_50_), where a shift of the curve to higher [Ca^2+^]_m_ represents a decrease in Ca^2+^ sensitivity [1].

The [Ca^2+^]_m_ is dependent on the amount of Ca^2+^ released with each activation and the amount of buffering of Ca^2+^ that is available [6]. We also know that the [Ca^2+^]_m_ is dependent on the frequency of activation [7] and that during intermittent stimulation at a fixed high-frequency, there is initially an increase in [Ca^2+^]_m_ in the early stages and then a decrease in the late stages of fatigue [8]. On the other hand, force decreases when [Ca^2+^]_m_ increases in the early stages, followed by a simultaneous decrease in both force and [Ca^2+^]_m_ in the late stages of fatigue. However, the pattern of change in [Ca^2+^]_m_ is not known for intermittent low-frequency stimulation in mammalian muscles, a situation that is more compatible with in vivo muscle activation.

Activation of muscle for locomotion and general movements typically does not require maximal activation. A common fatigue protocol that takes this into consideration was first used by Burke et al. [9] for the purpose of identifying contractile properties of specific muscle fiber types. Burke and colleagues stimulated the cat gastrocnemius muscle with 40 Hz trains lasting 300 ms at 1-s intervals. When whole muscle is stimulated in situ with this intermittent submaximal low-frequency pattern, there is a characteristic change in active force that typically includes a down, then up, then down change in active force [10]. Considering the multitude of factors that can contribute to increases and decreases in active force, it is of interest to see which factors dominate through this pattern of change in active low-frequency force. We know of several factors that can contribute to enhancement and depression (see below) of low-frequency force in response to intermittent stimulation. Some of these factors will influence the [Ca^2+^]_m_ and some will affect force by altering Ca^2+^ sensitivity, but either way, changes to both [Ca^2+^]_m_ and Ca^2+^ sensitivity will particularly affect low- as opposed to high-frequency force since contraction resulting from this stimulation lie on the steep portion of the force-[Ca^2+^]_m_ relationship.

An enhanced contractile response due to previous activity is thought to occur as a result of increased Ca^2+^ sensitivity [11] but could also result from increased [Ca^2+^]_m_. The mechanism of potentiation is generally thought to be dependent on increased Ca^2+^ sensitivity in association with regulatory light chain (RLC) phosphorylation. This RLC phosphorylation is thought to increase the rate of attachment of cross-bridges due to the increased mobility of the myosin heads [12]. This increase in probability of attachment results in a faster rate of rise in force and higher submaximal active force, at any given [Ca^2+^]_m_. This form of potentiation is effective up to a frequency of stimulation corresponding to that which yields 60% of maximal active force [10].

Ca^2+^ sensitivity is altered by factors that change cross-bridge kinetics, which can alter the number of simultaneous force-generating cross-bridges at submaximal [Ca^2+^]_m_. Common mechanisms of change in cross-bridge kinetics affecting Ca^2+^ sensitivity include temperature, pH, myosin RLC phosphorylation, [P_i_], and sarcomere length [13]. A decrease in pH which is often reported to occur during repeated stimulation can contribute to decreased Ca^2+^ sensitivity, but the level of acidosis observed in single fibers is minor due to lactate transporters and a relatively large extracellular volume [14]. Furthermore, the impact of acidosis in depressing active force is reduced at physiological temperatures, relative to room temperature and lower [15, 16]. Finally, an increase in [P_i_] has been suggested to decrease Ca^2+^ sensitivity, mainly during early fatigue [17]. In late fatigue, increased [P_i_] is thought to cause a decrease in Ca^2+^ release from the sarcoplasmic reticulum (SR). This occurs because P_i_ can migrate into the SR and when [P_i_] is high enough in relation to free [Ca^2+^] in the SR, a precipitate of Ca-P_i_ will result, which decreases the amount of Ca^2+^ available to be released [18].

Considering that potentiation due to RLC phosphorylation should occur early in a series of repeated submaximal contractions [11] and that changes in Ca^2+^ sensitivity due to acidosis will be attenuated at physiological temperature [16], it was hypothesized that sensitivity to Ca^2+^ will decrease briefly, then increase and that any fatigue-induced decline in active force will be primarily dependent on decreases in [Ca^2+^]_m._ It was also hypothesized that the fibers would fully recover force at high frequencies with persistent reduced force at low frequencies (low-frequency fatigue). Considering that most factors that depress Ca^2+^ sensitivity will recover within minutes [19, 20], it was hypothesized that persistent depression of active force at low but not high frequencies would be due to decreased [Ca^2+^]_m_.

## Methods

### Animal treatment

Healthy, adult female C57bl/6J (Janvier Labs, France) mice were used for this study. All mice were given standard care. Mice were killed by rapid cervical disarticulation and the whole flexor digitorum brevis (FDB) muscle was dissected to isolate single muscle fibers (see below).

### Solutions

Tyrode solution was used during dissection and for superfusion during the experiments [3]. The solution consists of the following (mM): 121 NaCl, 5.0 KCl, 1.8 CaCl_2_, 0.5 MgCl_2_, 0.4 NaH_2_PO_4_, 24.0 NaHCO_3_, 0.1 EDTA, 5.5 glucose, and 0.2% fetal calf serum. The solution was bubbled with 5% CO_2_–95% O_2_ to keep pH at 7.4. The temperature of the Tyrode solution in the tissue bath was kept at 32 °C throughout the procedures. Indo-1 (#0146, TEFLABS, Austin, TX, USA), a [Ca^2+^]_m_ indicator, and 5 mM caffeine were also used in the experiments [3].

### Dissection and fiber preparation

The FDB muscles from a mouse were dissected down to a single, intact fiber using the method described by Cheng and Westerblad [3]. Tendons were folded into aluminum T-clips, and suspended between two hooks: one affixed to a Kronex Technologies AE801 force transducer and one that allowed length adjustment. The contractile bath was transferred to the stage of an inverted fluorescence microscope (Nikon Instruments, Eclipse Ti-S) with constant flow of Tyrode solution.

The single fiber was set to optimal length, determined with sequential 100 Hz stimulation for 350 ms, and was lengthened or shortened between contractions until the greatest isometric force was obtained. Each fiber was then microinjected with a [Ca^2+^]_m_ indicator (< 100 μM), Indo-1, which was first dissolved in 150 mM KCl and 10 mM HEPES. Injection was followed by rest for 20–30 min to let the Indo-1 diffuse throughout the fiber. The fiber diameter was recorded as the average measured at both ends of the fiber. Diameter was used to calculate cross-sectional area, assuming a circular fiber. Excitation of Indo-1 was achieved by Xenon light source, passing through a monochromator to selectively illuminate the fiber at 360 nm. Emitted light was filtered and quantified at 405 ± 5 and 495 ± 5 nm with dual photomultiplier tubes (Photon Technology International). A FeliX32 Analysis Module (Photon Technology International) was used for data collection (sampling rate of 500 Hz) and for analysis.

### Standard protocol

To establish the control relationship between force and [Ca^2+^]_m_, each fiber (*n* = 12) was stimulated at increasing frequencies (15, 20, 30, 40, 50, 70, 100, 120, 150, 200 Hz) for 350 ms at 1-min intervals. A 5-min recovery period was allowed following the last contraction before the intermittent stimulation was initiated. In some cases, a longer rest interval was allowed because it was noticed that active force for 50 Hz stimulation was depressed relative to the value obtained during the above contractions. This extra rest (up to 30 min) was not sufficient to restore active force. An additional three fibers were tested with prolonged rest (5–10 min) between contractions at a reduced number of frequencies (30, 40 50, 70, 100, and 200 Hz). This was done to evaluate the impact of potentiation or fatigue affecting the control force-[Ca^2+^]_m_ relationship.

Intermittent contractions were elicited at 2-s intervals at a frequency of either 40 Hz (*n* = 8) or 50 Hz (*n* = 4) stimulation for 350 ms, whichever gave an active force closest to 50% of maximum force. After 2 min, the interval was changed to 1 s, if necessary, until force was < 40% of the starting force. Force and fluorescence were continuously recorded and were assessed every 10% of the time course. A 200 Hz stimulus for 350 ms was given immediately afterwards to determine maximal force and corresponding [Ca^2+^]_m_.

Recovery started immediately after the 200 Hz contraction. At 5, 15, 25, and 35 min of recovery, the fiber was stimulated at three increasing frequencies (30, 50, 200 Hz), each for 350 ms at 1-min intervals to allow for the periodic assessment of the force-[Ca^2+^]_m_ relationship during the recovery period. After the last stimulation given at 37 min, some of the fibers (*n* = 4) were superfused for 2 min with Tyrode solution containing 5 mM of caffeine, then stimulated at 200 Hz to assure maximal activation. After seeing four consecutive fibers producing similar force as when stimulated at 200 Hz at 37 min, the use of caffeine was stopped.

### Control protocol

Three fibers (*n* = 3) were tested as above, except they were allowed to rest for a duration of 3 min during the time the fiber would have been stimulated intermittently (intermittent contractions protocol). At the end of 3 min, the fiber was still stimulated at 200 Hz for 350 ms and recovery was monitored as above. This control protocol allowed the determination of the impact of the contractions during the recovery period on the recovery of active force.

## Analysis

### Quantification of active force

The force transducer was calibrated with known weights. Active force is presented as force per fiber cross-sectional area (kPa). Peak and passive force was measured for contractions at 10% (time) intervals during intermittent stimulation. Passive force was measured just before each contraction. Active force was calculated as peak force minus passive force. Active force will also be presented as relative force using the control 200 Hz contraction as denominator.

### Quantification of [Ca^***2***+^]

Free myoplasmic [Ca^2+^], ([Ca^2+^]_m_) was determined by measuring the ratio (*R*) of fluorescence at the two emission wavelengths (405 and 495 nm) after background subtraction [21]. The ratio, calculated as an average across the plateau of the measured response, was used in the following equation derived by Grynkiewicz and colleagues [22] to calculate [Ca^2+^]_m_:

1 $$ {\left[{\mathrm{Ca}}^{2+}\right]}_{\mathrm{m}}={\mathrm{K}}_{\mathrm{D}}\times \mathrm{\ss}\left(R-{R}_{min}\right)\left({R}_{max}-R\right)-1 $$

where *K*_D_ is the apparent dissociation constant of Indo-1 [22]; *R*_min_ is the ratio at very low [Ca^2+^]_m_ (obtained by injecting 0.5 M EGTA into the fibers to get resting [Ca^2+^]_m_); *R*_max_ is the ratio at saturating [Ca^2+^]_m_ (obtained by injecting 1 mM calcium into the fibers to allow high [Ca^2+^] _m_); and ß is estimated (see Eq. (2) with the ratio of the 495 nm signals at low and saturating [Ca^2+^]_m_ [22]. These values are found using the method described by Bakker and colleagues (1993).

2 $$ \mathrm{\ss}=\left(1\hbox{--} {mR}_{\mathrm{max}}\right)\times {\left(1\hbox{--} {mR}_{\mathrm{min}}\right)}^{-1} $$

Where *m* is the slope using linear regression from plotting 495 vs. 405 nm [23].

### The Force-[Ca^***2***+^]_m_ relationship

Once [Ca^2+^]_m_ was calculated for contractions at various frequencies, the force-[Ca^2+^]_m_ relationship can be plotted and Hill’s equation (Eq. (3) can be fit to the data (4):

3 $$ \mathrm{F}={\mathrm{F}}_{\mathrm{m}\mathrm{ax}}{{\left[{\mathrm{Ca}}^{2+}\left]{{}_{\mathrm{m}}}^{\mathrm{N}}/\Big({{\mathrm{Ca}}_{50}}^{\mathrm{N}}+\right[{\mathrm{Ca}}^{2+}\right]}_{\mathrm{m}}}^{\mathrm{N}}\Big) $$

where *F* is force, *F*_max_ is the force at saturating [Ca^2+^]_m_, Ca_50_ is [Ca^2+^]_m_ at 50% of *F*_max_, and *N* is a constant to describe steepness of the function. It was assumed that the contraction at 200 Hz gave saturating [Ca^2+^]_m_. The force-[Ca^2+^] curve for each fiber was used to compare changes in [Ca^2+^]_m_ during fatigue and recovery.

### Determination of Ca_50_

[Ca^2+^] at half the maximum force (Ca_50_) was one of the constants yielded by fitting the data to Eq. (3. This value was used as an estimate of the calcium sensitivity. An estimate of Ca_50_ was obtained at the beginning and end of the intermittent contractions, and at each measurement time during recovery. This estimate used the same Hill coefficient as the control force-[Ca^2+^] relationship, and Ca_50_ and *F*_max_ were adjusted to fit through the available data.

### Statistics

Data are presented as a mean ± SEM or SD as indicated and compared using one-way repeated measures ANOVA to evaluate the Ca_50_ values obtained at the following times: first contraction of intermittent stimulation, the end of fatigue, and at 5 and 15 min recovery times. It was noted that Ca_50_ did not change much after 15 min, so these times were compared without consideration of longer recovery times to reduce the number of comparisons for improved statistical power. The ANOVA was followed by multiple comparisons with the Tukey’s post-hoc analysis to detect significant differences over time. Results were considered significant when *p* < 0.05.

## Results

### Fiber characteristics

Average fiber diameter was 32.0 ± 4.4 μm. Maximal isometric force prior to intermittent contractions was 451 ± 60 kPa.

### Control force-frequency and force-[Ca^**2**+^]

**Fig. 1:**
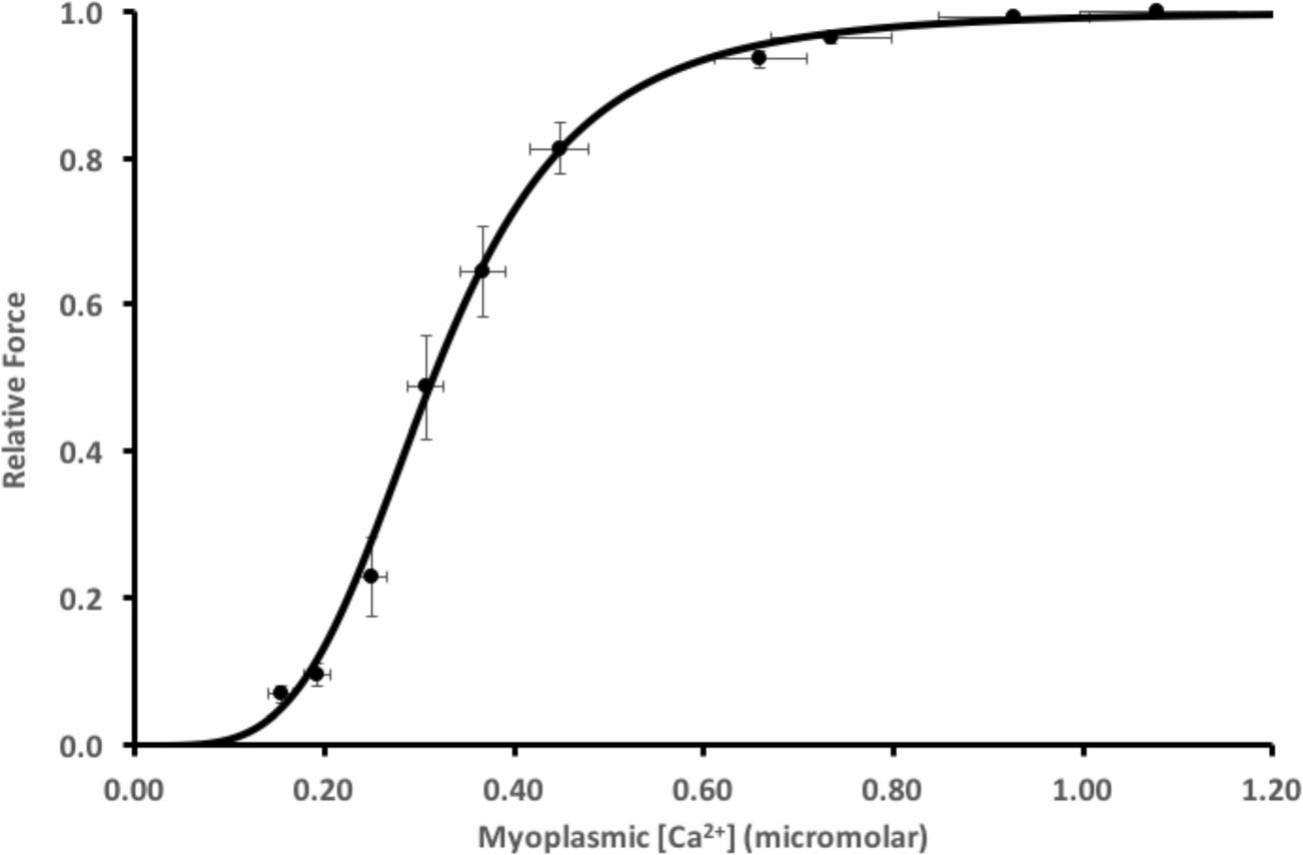
Mean ± SEM force-[Ca^2+^]_m_ relationship. Relative force with the myoplasmic free [Ca^2+^] for *n* = 12 fibers during increasing frequencies (15–200 Hz) stimulated every minute during the initial standard protocol. The line is best fit through the data with half-maximal [Ca^2+^]_m_ 3.14E-7M. Hill coefficient was 4.1

The force-frequency data obtained during the first part of the standard protocol gave a sigmoidal relationship between active force and [Ca^2+^]_m_ as illustrated in Fig. 1. The steepest part of the slope occurred between 30 and 70 Hz, where there was a substantial increase in force with small increases in frequency and corresponding small increases in [Ca^2+^]_m_. Fitting these data to Eq. (3 yielded a Ca_50_ of 0.304 μM. When we compared this control force-[Ca^2+^]_m_ curve to the first contraction of the intermittent stimulation protocol that followed, we noted that the first contraction occurred to the right of this control curve and at lower [Ca^2+^]_m_. This observation could be a result of potentiation of the original force-frequency data, and/or persistent fatigue resulting from completion of these contractions.

#### Initial potentiation caused by standard protocol

Additional experiments (*n* = 3) were completed to see if the control force-[Ca^2+^]_m_ relationship reflected fatigue and/or activity-dependent potentiation, explaining the difference in apparent Ca_50_. Fewer contractions at longer (5–10 min) intervals were obtained (30, 40, 50, 70, 100, and 200 Hz). When this was done, the first contraction of the intermittent contractions was closer to or superimposed on the control force-[Ca^2+^]_m_ relationship so we concluded that the control force-[Ca^2+^]_m_ relationship, obtained with 1-min intervals in the original fibers, reflected activity-dependent potentiation. For this reason, further comparisons during and following the intermittent contractions are between the apparent Ca_50_ for the first contraction of the intermittent contractions. This was assumed to represent the non-potentiated condition. The Ca_50_ for this contraction was obtained using the same maximum, minimum (fixed at zero), and slope for the control Hill equation. Ca_50_ was adjusted until the curve went through the value for the first contraction. The first contraction was consistently weaker than the control contraction at the same frequency. We attribute this to long-lasting fatigue resulting from the contractions used to establish the control force-[Ca^2+^]_m_ relationship.

### Intermittent stimulation

It took a fiber on average 77 ± 34 (mean ± SD) contractions to decrease force to 40% of initial force.

#### Staircase potentiation (phase 1 and 2)

**Fig. 2:**
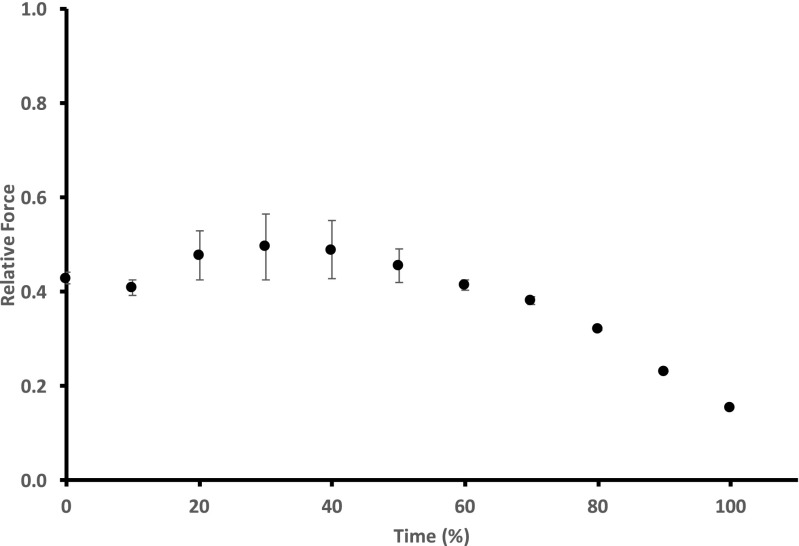
Time course for relative force during standard intermittent stimulation protocol. Active force typically decreased from the initial value (phase 1), then increased until 30% of time (phase 2). This was followed by decreasing active force (phase 3 and 4). Values are mean ± SEM (*n* = 12). Missing error bars are within the size of the symbol

During the time course of intermittent stimulation, expressed as percent of total time, an initial decrease in active force was observed (phase 1), followed by an increase in active force until about 30% into the duration (phase 2). Following this, there was a slow decline, which usually was not sufficient to reach the 40% force target within 2 min. This target was achieved shortly after changing the frequency of contractions to one per second. The initial down then up staircase potentiation is shown in Fig. 2, where force increases on average 27 kPa from the initial force (194 kPa). Not every fiber demonstrated the initial decrease in force when looking at the time course of fatigue in 10% increments. However, in every case of the standard protocol, there was a decrease in active force before the increase in force that typically peaked at ~ 30% of elapsed time.

#### Intermittent stimulation-induced fatigue (phase 3 and 4)

**Fig. 3:**
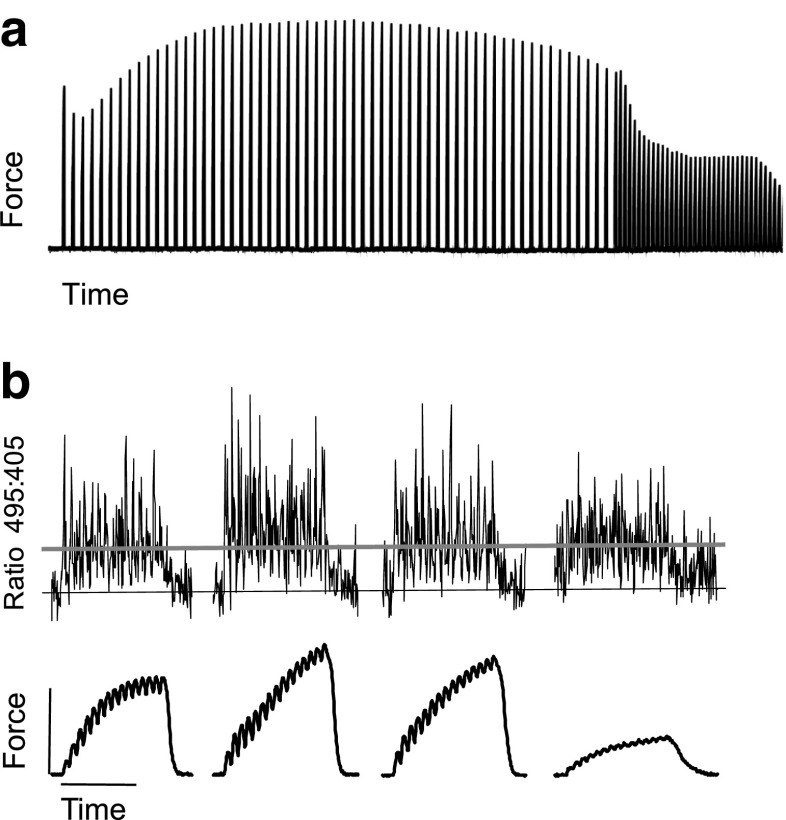
Time course of active force for one fiber. **a** Stimulated at 50 Hz every 2 s for 2 min, then every 1 s until force was < 40% of initial force. **b** Sample contractions on expanded time scale are shown illustrating [Ca^2+^]_m_ (upper tracing) and force (lower tracing) at start of intermittent stimulation, 40% of duration, 70% of duration, and end of intermittent stimulation where < 40% of initial force is reached, respectively. Horizontal black line corresponds with average resting myoplasmic free [Ca^2+^] for the first contraction. Horizontal gray line corresponds with average myoplasmic free [Ca^2+^] during the first contraction (0.3 μM). Vertical calibration bar is 200 kPa. Horizontal calibration bar is 200 ms

Following this peak, force decreased in two phases to 40% of initial force (phase 3 and 4, see below). Raw data for one fiber during the fatigue protocol is shown in Fig. 3, with examples of both force and [Ca^2+^]_m_ on an expanded time scale at different time points throughout the period of intermittent stimulation.

#### Changes in fusion index throughout intermittent stimulations

**Fig. 4:**
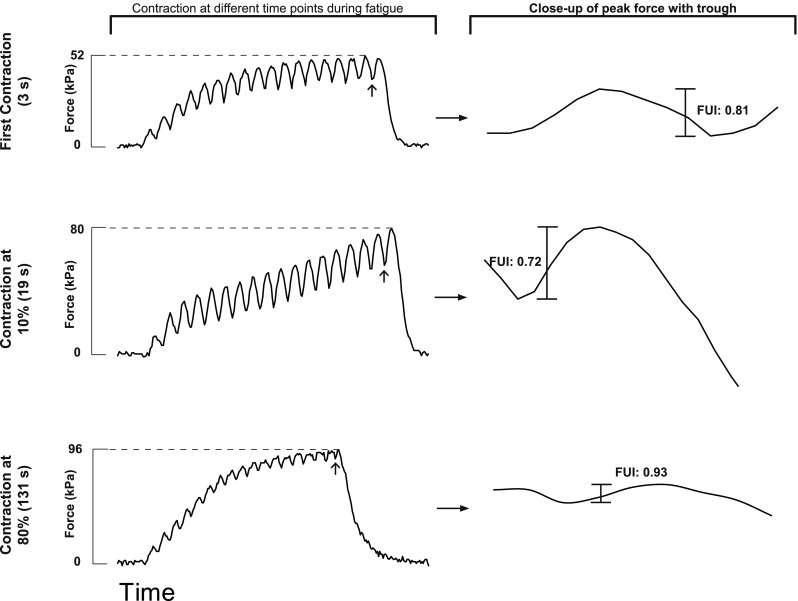
Sample estimation of fusion index (FUI) during intermittent contractions. The left column shows the force for 350 ms contractions at specific times during repetitive stimulation. The dotted horizontal line indicates peak force and the arrow points at the trough (minimum value) that was used to calculate the fusion index. The right column demonstrates a close-up view of the trough and peak for each contraction and the magnitude of fusion index

The fusion index (FUI) was calculated at 10% time increments throughout the time course of intermittent stimulation, to identify a potential mechanism for change in Ca^2+^ sensitivity. Accelerated relaxation is assumed to result in less summation of force for a given [Ca^2+^]_m_, reflecting a decreased Ca^2+^ sensitivity. Figure 4 shows sample contractions with the close-up of fusion index estimation at peak force for one fiber at three different time points during intermittent stimulation. Although there was a trend for fusion index to decrease by 10% of duration of the intermittent stimulation, then increase, there were no significant differences observed for the fusion index (data not presented).

#### A “P” pattern of changes in force and [Ca^***2***+^]_m_

**Fig. 5:**
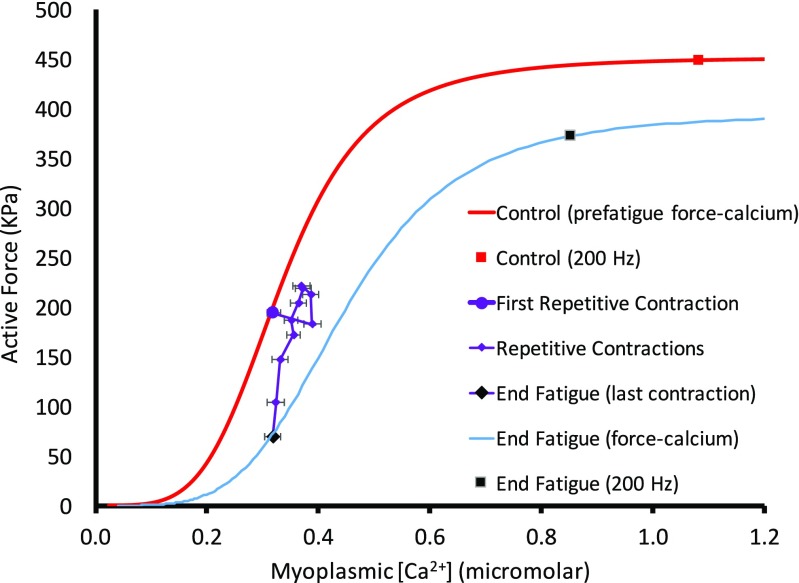
Force-[Ca^2+^]_m_ relationship during intermittent stimulation. Values during the intermittent stimulation (*n* = 12) are shown in purple, beginning with the large purple dot. The control force-[Ca^2+^]_m_ curve was fit through the first contraction and the control 200 Hz contraction, using the same Hill coefficient as the control force-[Ca^2+^] shown in Fig. 1. The large purple diamond indicates the last contraction of the intermittent stimulation. The black square is the average force-[Ca^2+^]_m_ value when stimulated at 200 Hz at the end of fatigue. The curve through these two values represents the force-[Ca^2+^]_m_ relationship at the end of intermittent contractions (Ca_50_ = 4.65E-7M)

From the first contraction to the end of the intermittent stimulation, the average [Ca^2+^]_m_ and force appeared to change in a “P”-shaped pattern (Fig. 5). Initially, force decreased while [Ca^2+^]_m_ increased (phase 1). From here, force increased first without a change in [Ca^2+^]_m_ then with a corresponding decrease in [Ca^2+^]_m_ (phase 2). Following the potentiation, force decreased, first in parallel with the initial force-[Ca^2+^]_m_ relationship (phase 3), then without further change in [Ca^2+^]_m_ (phase 4). Considering the small change in [Ca^2+^]_m_ during phase 3, we evaluated the change to see if it reached statistical significance. A paired *t* test confirmed that this small change in [Ca^2+^]_m_ from 3.722E-7 ± 5.5E-8M at 30% time to 3.31E-7 ± 4.7E-8M (mean ± SE) at 80% time was significant (*p* < 0.001). However, there was not a difference in average tetanic [Ca^2+^]_m_ when comparing the start (3.2E-7M) and the end (3.2E-7M) of the intermittent contractions.

#### Force-[Ca^***2***+^]_m_ curves from start to end of intermittent stimulations

**Fig. 6:**
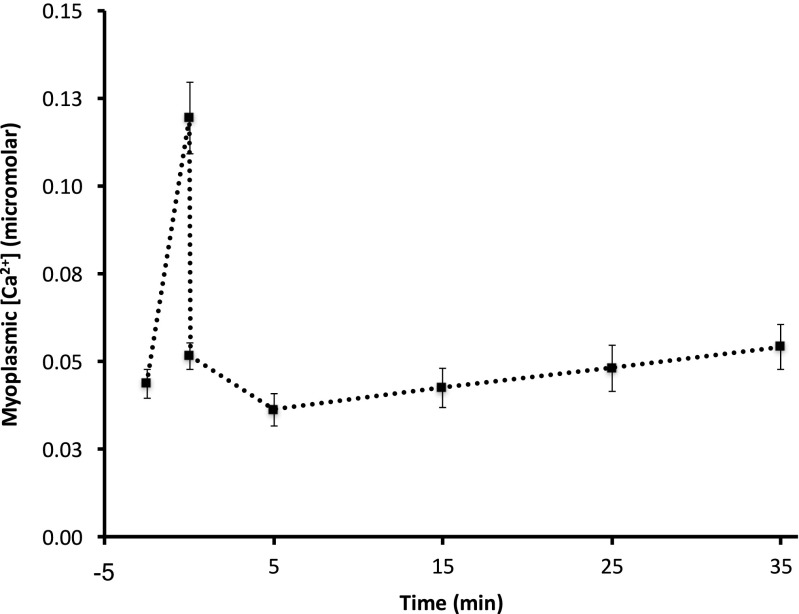
Resting myoplasmic free [Ca^2+^]_m_. Concentrations shown were measured prior to first and last of the intermittent contractions, the 200 Hz contraction at end of intermittent stimulation and contractions obtained throughout 35-min recovery. Clearly resting [Ca^2+^]_m_ increased during the intermittent stimulation, but recovered between the end of this stimulation and the 200 Hz contraction (within seconds). Vertical bars represent SEM

Two force-[Ca^2+^]_m_ curves have been superimposed on the data in Fig. 5: one to illustrate the initial conditions and one to show how these intermittent contractions have altered the relationship between force and [Ca^2+^]_m_ by the time the target force had been reached (< 40% of initial force). This latter curve was produced using the same Hill coefficient as the initial curve, but changing Ca_50_ and the maximal value. This maximal value for active force was obtained by stimulation at 200 Hz as soon as the target force was reached. Half-maximal [Ca^2+^]_m_ had increased to 3.45E-7M. Resting [Ca^2+^]_m_ increased throughout the period of intermittent contractions, but recovered within seconds when stimulation was stopped (see Fig. 6). There was no indication that this affected resting force.

### Recovery phase

**Fig. 7:**
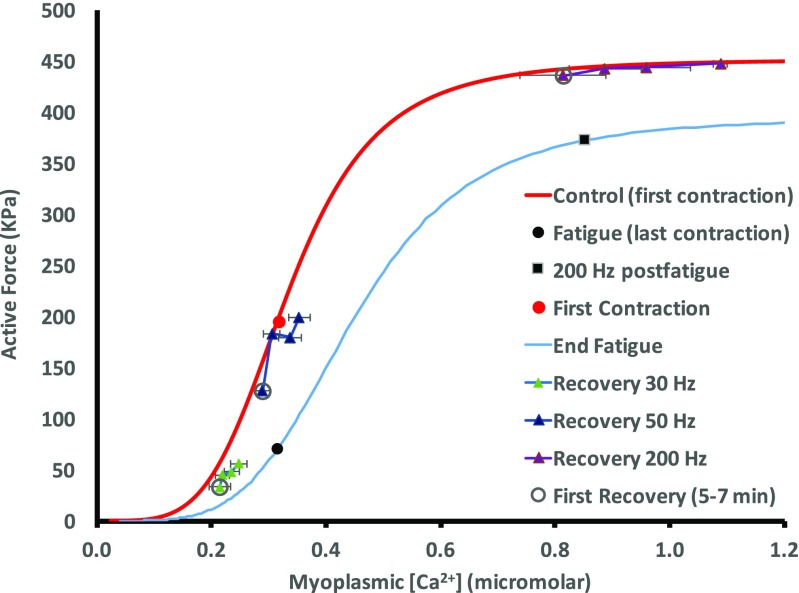
Force-[Ca^2+^]_m_ during recovery. Contractions at 30, 50, and 200 Hz were obtained during recovery at 5, 15, 25, and 35 min. First recovery values were measured at 5–7 min (open circles). The prefatigue force-[Ca^2+^]_m_ curve (red line) passes through the first contraction of the intermittent stimulation (red circle) for reference. Force and [Ca^2+^]_m_ tended to increase throughout recovery from 5 to 35 min. Data points represent the mean of *n* = 12. Horizontal bars represent SEM

During recovery, force and [Ca^2+^]_m_ at 30 and 50 Hz exhibited persistent fatigue (see Fig. 7). Force and [Ca^2+^]_m_ of these contractions continued to increase from 5 to 35 min but did not fully recover to prefatigue force. The 200 Hz force recovered to prefatigue condition by 5 min of recovery with [Ca^2+^]_m_ increasing throughout the recovery period without further change in maximum force.

### Control experiment

**Fig. 8:**
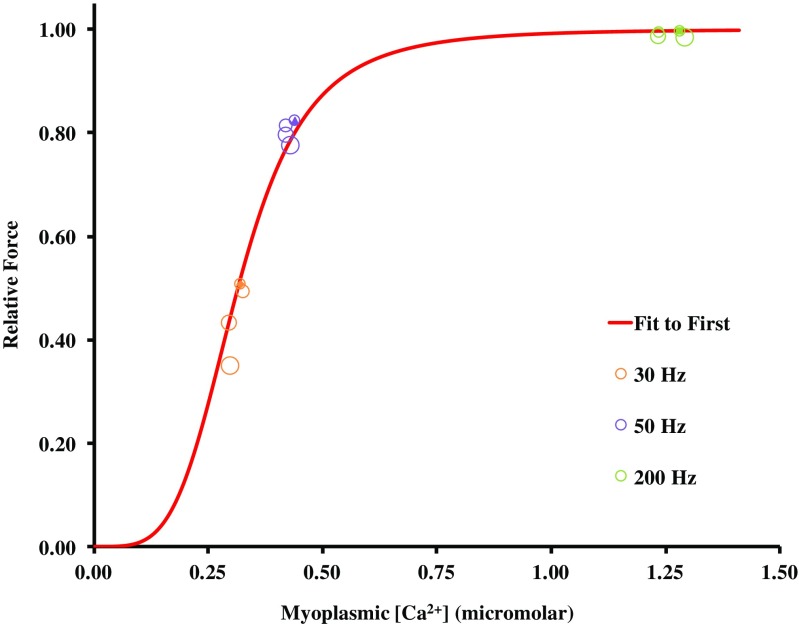
Force-[Ca^2+^]_m_ relationship during recovery without prior intermittent stimulation. Relative active force and [Ca^2+^]_m_ at 5, 15, 25, and 35 min representing recovery (open circles) without prior intermittent stimulation. Values are compared to control force-[Ca^2+^]_m_ relationship (red line) as in Figs. 5 and 7. Circles from small to large demonstrate values from 5 to 35 min. Data points presented as the mean of *n* = 3

The control experiment included the recovery contractions without prior intermittent contractions. The results of this experiment are shown in Fig. 8. It can be seen here that these “recovery” contractions progressively decreased in amplitude, particularly at the low frequencies. These control tests were completed to evaluate the impact of collecting these contractions on our assessment of recovery. These results indicate that the recovery contractions would have slowed the actual recovery following the intermittent contractions. In the experiments with 5–10 min rest between the control contractions at different frequencies, recovery was tested only at 35–37 min. In these experiments, active force at low frequency was still depressed relative to high-frequency force, demonstrating that persistent low-frequency fatigue was not simply a consequence of regular monitoring of contractions during the recovery period.

### Changes in Ca_50_ from prefatigue until end of recovery

**Fig. 9:**
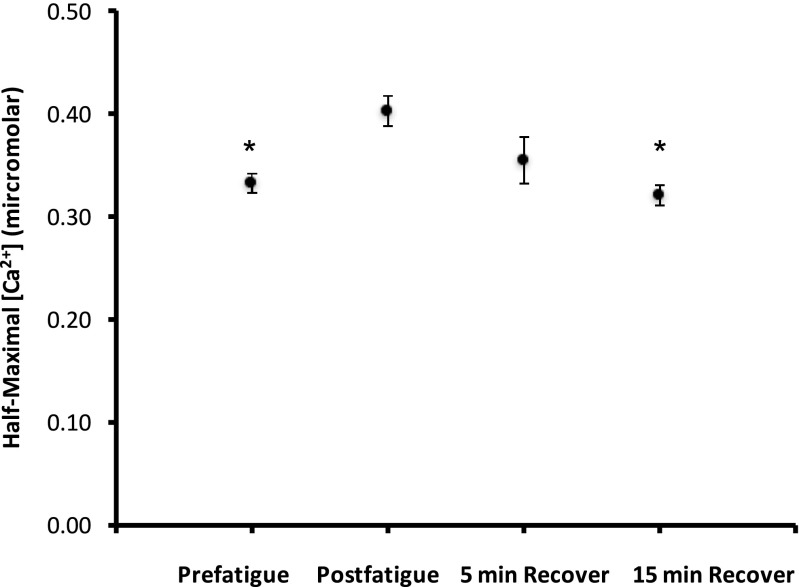
Ca_50_ values before and after intermittent contractions. The average ± SEM Ca_50_ for prefatigue (through first contraction), end of fatigue (last contraction), 5 min recovery, and 15 min recovery. Asterisk shows significant difference when compared to postfatigue (*p* < 0.05). Value at 5 min recovery was not different from any other values (*n* = 12)

The Ca_50_ was compared across four times: prefatigue, postfatigue, 5 min of recovery, and 15 min of recovery. As seen in Fig. 9, a significant effect was seen (*p* < 0.001), with post-hoc analysis showing a significant difference of Ca_50_ between start of fatigue and end of fatigue (*p* = 0.001). No significant difference was found when comparing Ca_50_ prefatigue to Ca_50_ at 5 and 15 min (*p* > 0.5). Values later in fatigue were not greater than the values observed at 5 min (data not shown).

#### Changes in sarcomere length

Sarcomere length was determined (*n* = 3) at the beginning of the test and again at the end to see if a decrease in force or change in Ca^2+^ sensitivity occur as a consequence of shortening of sarcomeres which could be a consequence of tendon stretch or repositioning of the aluminum clips. There was only a 0.1 μm shortening in the three fibers from start of test to the end (2.5, 2.9, 2.9 μm to 2.4, 2.8, 2.8 μm respectively). This length change would not be expected to decrease active force in a mouse fiber in which optimal length of active force is achieved at a plateau from 2.4 to 2.8 μm [24].

## Discussion

The key observations of this study during fatigue and recovery from submaximal intermittent contractions are (1) [Ca^2+^]_m_ increased then decreased during intermittent submaximal contractions; (2) Ca_50_ increased then decreased and finally increased again during intermittent submaximal contractions; (3) depression of force at the end of the intermittent contractions was primarily due to decreased Ca^2+^ sensitivity, but this recovered quickly and low-frequency fatigue (LFF) persisted into the recovery period due to low [Ca^2+^]_m_; and (4) only small changes in Ca_50_ were observed during the intermittent contractions. Each of these observations will be discussed below.

### Changes of force and Ca^**2**+^ during early intermittent stimulation (phase 1 and 2)

It was observed that the fibers were seemingly already “fatigued” before the intermittent contraction protocol started. The first measurement of force was lower than the force measured at the same frequency in the prefatigue force-frequency data and the first contraction was to the right of the control force-[Ca^2+^]_m_ relationship. A potential reason for this lower force is that the measure of force at 40 or 50 Hz in the force-frequency data was actually potentiated from previous stimulations (15, 20, 30 Hz), because the intervals between these contractions were only 1 min. Additional experiments confirmed this theory as the apparent force-[Ca^2+^]_m_ relationship through the first contraction of the intermittent stimulation was very close to that of the force-frequency data when the force-frequency data were collected with intervals of at least 5 min. However, the active force for the first contraction of the intermittent stimulation was still suppressed relative to that obtained with the 5-min intervals due to lower [Ca^2+^]_m_. Apparently, eliciting the contractions for the control force-[Ca^2+^]_m_ relationship already induces some long-lasting fatigue.

Early in the period of intermittent contractions (0–10% of duration), active force decreased (see Figs. 2 and 3), or remained fairly constant. In association with the initial decline, and before active force increased, there was an increase in [Ca^2+^]_m_ (evident in Fig. 5). Assuming maximal force has not yet changed, this increase in [Ca^2+^]_m_, with no increase in active force must represent a decrease in Ca^2+^ sensitivity. This increase in [Ca^2+^]_m_ while Ca^2+^ sensitivity decreased is similar to the change reported with maximal stimulation [8]. The mechanism of the decreased Ca^2+^ sensitivity is not known, but could relate to increased [P_i_] which has been identified previously as a factor decreasing Ca^2+^ sensitivity [25, 26]. Research has indicated that acidosis does not contribute to decreased Ca^2+^ sensitivity when physiological temperatures are used [27]. It is important to realize that an increase in [Ca^2+^]_m_ observed here is not evidence of increased SR Ca^2+^ release. Ca^2+^ is strongly buffered in skeletal muscle. During repeated contractions, these buffers will become progressively occupied. With decreased availability of buffers, less of the released Ca^2+^ will be bound, so more will remain free, creating a higher free [Ca^2+^]_m_.

Between 10 and 30% of total time (phase two), the fibers generally increased in force while [Ca^2+^]_m_ remained constant then decreased (Fig. 5). A similar down (phase 1) then up (phase 2) staircase response was reported by MacIntosh and Willis [10], with intermittent stimulation at 50, 60, and 70 Hz in rat whole gastrocnemius muscle in situ. It has historically been accepted that the positive response during a staircase of this nature is the result of increased RLC phosphorylation [11], which is known to increase Ca^2+^ sensitivity. Remarkably, there was no evidence of an increase in Ca^2+^ sensitivity (shift to the left for the force-[Ca^2+^]_m_ relationship) beyond the position of the initial contraction during the intermittent stimulation, as would have been expected during the staircase response consistently observed in our experiments. The change in active force during staircase is partly due to an increased Ca^2+^ sensitivity, so this mechanism would be consistent with RLC phosphorylation, but this change occurs relative to the already decreased Ca^2+^ sensitivity. This small relative change in Ca^2+^ sensitivity can only be explained by coexistence of a mechanism in the opposite direction [28]; some factor was decreasing Ca^2+^ sensitivity while RLC phosphorylation and possibly S-glutathionylation of troponin I [29] increased Ca^2+^ sensitivity. This combination of factors increasing and decreasing Ca^2+^ sensitivity has probably contributed to the fact that we observed only minor changes in Ca_50_ in this study (observation 4 above).

### Changes in [Ca^**2**+^]_m_ and Ca_50_ during decline in active force (phase 3 and 4)

From the peak of the staircase response (~ 30% time in Fig. 2), force continually decreased. The decrease in active force occurred initially (phase 3) in parallel with the control force-[Ca^2+^]_m_ relationship (Fig. 5), indicating that the decline in active force was primarily due to decreasing [Ca^2+^]_m_ with no change in Ca_50_. A decrease in [Ca^2+^]_m_ would require decreased Ca^2+^ release since increasing occupation of Ca^2+^ buffers would otherwise result in greater [Ca^2+^]_m_ if release remained the same. It should be kept in mind that SR Ca^2+^ release was not actually measured in this study. Dutka and Lamb [30] proposed that the decrease in [Ca^2+^]_m_ during late fatigue is due, in part, to the combination of reduced [ATP] with increased [Mg^2+^], inhibiting RyR activation thereby decreasing release of Ca^2+^. The reduced [ATP] may be a consequence of intramuscular glycogen depletion. Glycogen depletion is also known to cause decreased SR Ca^2+^ release [31, 32]. Fryer and colleagues [33] proposed another mechanism of reducing Ca^2+^ release is due to precipitation of Ca^2+^ and P_i_ in the SR, reducing the concentration of free Ca^2+^ available to be released. This mechanism has been supported by others [18]. It is possible that all of these mechanisms were effective, yet [Ca^2+^]_m_ was not depressed by much because more of the Ca^2+^ buffers were occupied, allowing a higher free [Ca^2+^]_m_ than would otherwise be expected.

In spite of possible factors impairing Ca^2+^ release, the final decrease in active force (phase 4) occurred with no further change in [Ca^2+^]_m,_ indicating that the decline was due to a decrease in Ca^2+^ sensitivity, possibly in combination with a decrease in maximal force, which was evident at the end of the period of intermittent contractions (Fig. 5). In this respect, the fatigue observed with submaximal contractions is different from that reported for intermittent high-frequency contractions. Westerblad and Allen [5] observed a late decrease in force that corresponded with a decrease in [Ca^2+^]_m_ when stimulated with repeated maximal tetani.

[Ca^2+^]_m_ at the end of the intermittent contractions observed here was not less than that for the first contraction (Fig. 5). Therefore, the final phase of the decrease in active force was entirely due to decreases in Ca^2+^ sensitivity and/or maximal force. This occurred in spite of the likelihood that RLC phosphorylation and possibly other potentiating factors, would be expected to persist at this time. This observation emphasizes the simultaneous impact of fatigue and potentiation [28]. Apparently, the mechanisms decreasing Ca^2+^ sensitivity were more effective than the mechanisms increasing Ca^2+^ sensitivity. Two possible mechanisms that are thought to decrease Ca^2+^ sensitivity include altered cross-bridge kinetics by increased [P_i_] [34] and/or increased production of reactive oxygen-nitrogen species [35, 36]. It should be noted that a decrease in force generating capacity will contribute to an apparent shift to the right in the force-[Ca^2+^]_m_ relationship. However, this mechanism will not affect Ca^2+^ sensitivity.

### Slowed relaxation

The fusion index of a contraction can be a good indication of the rate of relaxation (Fig. 4). It has been observed that a higher fusion index corresponds with a prolonged half-relaxation time [37]. A slowed relaxation time is typically seen during intermittent contractions leading to fatigue, and contributes to an increase in Ca^2+^ sensitivity. The mechanism associated with this increase in Ca^2+^ sensitivity can be due to either the slowing of Ca^2+^ dissociating from troponin C, or the slowing of detachment of cross-bridges [38]. The lowest FUI on average occurred at 10% of the time course of intermittent contractions and FUI increased from 30 to 80% of the time course of fatigue. However, differences in FUI were not significant. The reported changes in Ca^2+^ sensitivity during the intermittent contractions were not likely associated with mechanisms altering the FUI.

### Resting [Ca^**2**+^]_m_ during fatigue

The resting [Ca^2+^]_m_ during the intermittent stimulation demonstrates a linear relationship over time, increasing [Ca^2+^]_m_ from start to end of these contractions (Fig. 6). Westerblad and Allen [5] also observed an increase in resting [Ca^2+^]_m_ throughout their sequence of fatiguing maximal stimulation. This increase can be due to either increased leakage of Ca^2+^ from the SR or due to slowed Ca^2+^ re-uptake into the sarcoplasmic reticulum. The subcellular distribution of [Ca^2+^]_m_ was not measured during this study, preventing a more precise description of the mechanism for this increase in resting [Ca^2+^]_m_. It is interesting to note that the increase in resting [Ca^2+^]_m_ could have caused persistent active force but the decrease in Ca^2+^ sensitivity prevented this during this period of elevated resting [Ca^2+^]_m_. Resting [Ca^2+^]_m_ returned to the prefatigue level by the time the 200 Hz contraction was elicited.

### Ca_50_ during recovery

After the fibers were fatigued, force and [Ca^2+^]_m_ were measured periodically during a 35-min period of recovery (Fig. 7). Ca_50_ values were calculated at early recovery times (5 and 15 min) and compared to the Ca_50_ from prefatigue and the end of intermittent stimulation (postfatigue). Ca^2+^ sensitivity had decreased at the end of the intermittent contractions but had recovered such that Ca_50_ was not different from prefatigue values at both 5 and 15 min of recovery. During intense intermittent stimulation [35], a ROS-related reduction in Ca^2+^ sensitivity can be observed during recovery since ROS generation increases with increasing contraction intensity [39]. The results of the current study, however, suggest that a ROS-induced alteration in Ca^2+^ sensitivity following low-frequency contractions was unlikely since Ca_50_ was not affected during recovery. Thus, lower [Ca^2+^]_m_ was the primary mechanism for the persistent LFF following low-frequency contractions.

LFF was also observed by Westerblad et al. [2] and Chin and Allen [40] and it was suggested that the persistent fatigue during recovery was caused by decreased [Ca^2+^]_m,_ not decreased Ca^2+^ sensitivity [7, 40]. Allen et al. [41] suggested that the mechanism for decreased [Ca^2+^]_m_ in LFF is due to disrupted excitation-contraction coupling [41]. More specifically, the decrease in [Ca^2+^]_m_ is likely due to impaired Ca^2+^ release from the SR as the amount of free [Ca^2+^]_m_ in the SR should not be affected after a few minutes of recovery. Further research will be required to confirm this possibility.

Some researchers have indicated that increased ROS during repeated stimulation can inhibit the RyR, disrupting the release of Ca^2+^ and/or that depletion of glycogen can cause decreased Ca^2+^ release [31, 36, 42–44]. Four fibers were stimulated at 200 Hz with added caffeine at the end of testing where maximal force was observed (101 ± 2% of force measured at 200 Hz prefatigue), confirming that the contractile proteins were not disrupted or impaired and the amount of free [Ca^2+^]_m_ in the SR was not affected. This was also seen by Westerblad et al. [2]. Cheng et al. [31] have shown that recovery from low-frequency fatigue corresponds with glycogen resynthesis and recovery can be arrested by preventing glycogen resynthesis. Figure 7 demonstrates the slow increase in [Ca^2+^]_m_ at 30, 50, and 200 Hz during the recovery period. In agreement with Westerblad et al. [2], the decrease in force with slightly reduced [Ca^2+^]_m_ observed throughout recovery indicates that decreased [Ca^2+^]_m_ was the likely cause of low-frequency fatigue. This is consistent with the absence of change in Ca^2+^ sensitivity.

### Novel findings

Novel findings of this study include identifying that the fatigue-induced changes in force and [Ca^2+^]_m_ differ during low-frequency compared with high-frequency intermittent stimulation reported previously. The resulting P-shaped change of force and [Ca^2+^]_m_ has not been observed during high-frequency stimulation probably because low-frequency forces, which lie on the steepest portion of the force-[Ca^2+^]_m_ relationship, are particularly sensitive to alterations in SR Ca^2+^ release and myofibrillar Ca^2+^ sensitivity that can increase as well as decrease force. Another novel observation was that force depression during the recovery period after fatigue induced by low-frequency stimulation was completely explained by impaired SR Ca^2+^ release, which is different from the consequences of high-frequency stimulation where impaired myofibrillar Ca^2+^ sensitivity also contributes to the persistent impairment of low-frequency force production. Thus, the mechanisms underlying fatigue and recovery of force generation are clearly task dependent.

### Limitations

The measurement of [Ca^2+^]_m_ during intermittent submaximal contractions and subsequent recovery permits assessment of the primary mechanisms of fatigue. However, the underlying cause of the change in [Ca^2+^]_m_ and Ca_50_ has not been determined. The potential impact of changes in P_i_ concentration and Ca^2+^ release represent best guesses at these mechanisms. The use of Indo-1 for detection of [Ca^2+^]_m_ cannot detect quickly changing concentrations, but is suitable for resting and average concentrations during tetanic stimulation [21].

## Conclusion

In conclusion, results suggest that the mechanism of fatigue during submaximal intermittent contractions is a combination of changes to both [Ca^2+^]_m_ and Ca^2+^ sensitivity. Early in the intermittent contractions, the reduced force is due to decreased Ca^2+^ sensitivity. This is followed by an increased Ca^2+^ sensitivity while force potentiates above the original force, creating the loop part of the “P” force-[Ca^2+^]_m_ pattern. During late fatigue, the decline in force is first due to decreased [Ca^2+^]_m_, changing to a decreased Ca^2+^ sensitivity, both of which create the shaft part of the “P” pattern. During recovery, the persistent depression of low-frequency force was completely explained by decreased [Ca^2+^]_m_. Clearly, there are different patterns of change in the relationship between force and Ca^2+^ during intermittent low-frequency contractions than have been reported for high-frequency contractions.
